# Identification and Potential Use of Clusters of Patients With Colorectal Cancer and Patients With Prostate Cancer in Clinical Practice: Explorative Mixed Methods Study

**DOI:** 10.2196/42908

**Published:** 2022-12-27

**Authors:** Maik J M Beuken, Iris M Kanera, Nicole Paulina Maria Ezendam, Susy Braun, Martijn Zoet

**Affiliations:** 1 Faculty of Financial Management Research Centre for Future Proof Financials Zuyd University of Applied Sciences Sittard Netherlands; 2 Faculty of Health School of Physiotherapy Zuyd University of Applied Sciences Heerlen Netherlands; 3 Department of Research Netherlands Comprehensive Cancer Organization Utrecht Netherlands; 4 Center of Research on Psychology in Somatic Diseases Department of Medical and Clinical Psychology Tilburg University Tilburg Netherlands

**Keywords:** colorectal cancer, prostate cancer, referral to aftercare, patient clusters, cluster analysis, K-means cluster algorithm, multiple-factor analysis, expert panel group interviews, interview, cancer, patient, usability, clinical, colorectal, recovery

## Abstract

**Background:**

A steady increase in colorectal and prostate cancer survivors and patients with these cancers is expected in the upcoming years. As a result of primary cancer treatments, patients have numerous additional complaints, increasing the need for cancer aftercare. However, referrals to appropriate cancer aftercare remain inadequate, despite a wide range of aftercare options. Caregivers and patients often do not know which aftercare is the most appropriate for the individual patient. Since characteristics and complaints of patients within a diagnosis group may differ, predefined patient clusters could provide substantive and efficient support for professionals in the conversation about aftercare. By using advanced data analysis methods, clusters of patients who are different from one another within a diagnosis group can be identified.

**Objective:**

This study had a 2-fold objective: (1) to identify, visualize, and describe potential patient clusters within the colorectal and prostate cancer population and (2) to explore the potential usability of these clusters in clinical practice.

**Methods:**

First, we used cross-sectional data from patients with colorectal cancer and patients with prostate cancer provided by the population-based PROFILES (Patient-Reported Outcomes Following Initial Treatment and Long-Term Evaluation of Survivorship) registry, which were originally collected between 2008 and 2012. To identify and visualize different clusters among the 2 patient populations, we conducted cluster analyses by applying the K-means algorithm and multiple-factor analyses. Second, in a qualitative study, we presented the patient clusters to patients with prostate, patients with colorectal cancer, and oncology professionals. To assess the usability of these clusters, we held expert panel group interviews. The interviews were video recorded and transcribed. Three researchers independently performed content-directed data analyses to understand and describe the qualitative data. Quotes illustrate the most important results.

**Results:**

We identified 3 patient clusters among colorectal cancer cases (n=3989) and 5 patient clusters among prostate cancer cases (n=696), which were described in tabular form. Patient experts (6/8, 75%) and professional experts (17/20, 85%) recognized the patient clustering based on distinguishing variables. However, the tabular form was evaluated as less applicable in clinical practice. Instead, the experts suggested the development of a conversation tool (eg, decision tree) to guide professionals through the hierarchy of variables. In addition, participants suggested that information about possible aftercare initiatives should be offered and integrated. This would also ensure a good overview and seemed to be a precondition for finding suitable aftercare.

**Conclusions:**

This study demonstrates that a fully data-driven approach can be used to identify distinguishable and recognizable (ie, in routine care) patient clusters in large data sets within cancer populations. Patient clusters can be a source of support for health professionals in the aftercare conversation. These clusters, when integrated into a smart digital conversation and referral tool, might be an opportunity to improve referral to cancer aftercare.

**Trial Registration:**

Netherlands Trial Register NL9226; https://trialsearch.who.int/Trial2.aspx?TrialID=NL9226

## Introduction

Cancer represents one of the major global health care problems. In 2020, the incidence of all forms of cancer was higher than 18 million cases worldwide. Colorectal and prostate cancer are 2 of the top 4 most diagnosed cancers [1]. In 2020, approximately 11,500 new cases of colorectal cancer and over 12,000 new cases of prostate cancer were reported in the Netherlands alone [2]. Within the next 2 decades, these annual numbers in the Netherlands are expected to increase by 35% for colorectal cancer cases and 25% for prostate cancer cases. Fortunately, due to improved diagnostics and treatments, the 10-year survival rate of prostate cancer has risen to above 70% and that of colorectal cancer to almost 60% [2].

Cancer survivors are at a higher risk of developing new forms of cancer and comorbidities, as well as long-term physical, lifestyle, and psychosocial problems and difficulties with work. Consequently, an increasing number of survivors require information and support [3,4]. Earlier research has indicated that adequate cancer aftercare can support survivors to increase and maintain health, well-being, and quality of life [5-7].

Currently, cancer care in Dutch hospitals focuses on treatment by medical specialists, who do not always refer to additional (after)care interventions that match patients' wishes and needs [8]. Especially after intensive treatments, some patients do not know what to expect regarding their further recovery and how to resume a normal life. The general practitioner (GP) or specialist nurse in general practice could be in a position to monitor recovery and initiate a referral to appropriate aftercare tailored to survivors' needs. However, due to the lack of time, resources, and knowledge, family physicians also experience barriers to providing cancer aftercare. Moreover, patients may not perceive GPs and nurses as experts in cancer aftercare [9].

The European Academy of Cancer Sciences and other European organizations and cancer centers have emphasized the urgency of tailored aftercare in their published research agenda to reduce the major cancer burden and improve health-related quality of life by promoting cost-effective and evidence-based best practices in cancer prevention, treatment, care, and aftercare [10]. One of their recommendations for psychosocial oncology, rehabilitation, and survivorship research is to develop tools to enhance communication with patients and shared decision-making, such as the development and testing of decision aids for selecting aftercare. These are also key points in the recently published Dutch National Cancer and Life Action Plan [8].

In this paper, we explore the potential benefits of and barriers to patient clusters within the referral process. Referral to an aftercare option might be more appropriate and faster if distinguishing characteristics are considered. Clustering patient groups with similar characteristics may provide substantive and efficient support for professionals in the conversation about aftercare. Recently, researchers have explored new approaches to data analysis to identify patient clusters. Nicolet et al [11] used a clustering technique to highlight clinically relevant clusters and eventually identify profiles that use more health care and incur higher costs. The K-means algorithm is more commonly used to classify patients into clusters. Elbattah et al [12] used K-means to cluster elderly patients into groups. The K-means clustering technique is also frequently used in studies that focus on clustering patients with cancer. Florenca et al [13] recently used K-means to identify similar profiles of patients with colorectal cancer based on risk factors, and Kim et al [14] applied K-means to classify patients with breast cancer based on their level of adherence. In this study, we consider clustering variables related to long-term problems after cancer, including sociodemographic, health-related, psychosocial, lifestyle factors, and quality of life variables. The use of K-means to cluster patients into profiles based on a wide range of variables and the use of the multiple factor analysis (MFA) to interpret these profiles is a different approach from the aforementioned studies. To verify this fully data-driven approach in daily practice, we combined it with a qualitative evaluation among professionals and former and current patients with cancer.

This study had a 2-fold aim: (1) to identify, visualize, and describe potential patient clusters within colorectal and prostate cancer populations and (2) to explore the potential usability of these patient clusters in clinical practice.

## Methods

### Overview

This section is organized as follows. In part 1, we address the first aim of identifying, visualizing, and describing patient clusters. The clinical usability of the identified patient clusters is reported in part 2.

### Ethics Approval

This study was carried out in accordance with the ethics committee Medisch Ethische ToetsingsCommissie Zuyderland at Zuyd Hogeschool (METCZ20200203). Ethical approval was obtained for the study samples from the certified medical ethics committee Maxima Medisch Centrum (0822). Informed consent was obtained from all individual participants included in the study, including consent for secondary data analysis. Data from the PROFILES (Patient-Reported Outcomes Following Initial Treatment and Long-term Evaluation of Survivorship) registry were used. These data are freely available for noncommercial scientific research, subject to the study question, privacy and confidentiality restrictions, and registration [15]. Data were deidentified and pseudonymized. Patients did not receive any financial compensation for study participation.

### Part 1: Patient Clusters

#### Design

As previously mentioned, to identify patient clusters, we used cross-sectional data from the population-based PROFILES registry [16], which collects patient-reported outcomes in a large cohort to study the psychosocial and physical impacts of cancer and its treatment.

#### Study Population

From the PROFILES registry, we included 2 patient samples with colorectal cancer collected between 2008 and 2011 and 1 patient sample with prostate cancer collected between 2011 and 2012. A detailed description of the data collection method within the PROFILES registry has been reported elsewhere [16]. A population-based sampling frame was used, where patients were selected from the Netherlands Cancer Registry from a selected set of participating hospitals. In this study, we used the entire data set without sampling from it. Patients needed to be able to complete a Dutch questionnaire and be 18 years or older. Patients were invited by their treating oncology surgeon (colorectal cancer) or urologist (prostate cancer). There were no other inclusion or exclusion criteria to assure the population-based sampling.

#### Measurements

For the cluster analysis, we used all available variables from the PROFILES data set provided, including the following self-reported measures: sociodemographic information (regarding marital status, educational level, and employment), socioeconomic status [17], and emotional and cognitive functioning. We included all available patient-related outcome measurements in Multimedia Appendix 1 [16,18-26].

#### Statistical Analyses

##### Handling Data for Data Analysis

We conducted the data analyses on colorectal and prostate cancer samples separately. We merged both colorectal cancer samples and assessed all data for aberrant measurement data, missing data, and outliers.

Missing data were imputed by using the K-nearest neighbor (KNN) method (VIM package) [27]. All variables were used to impute missing values. In the KNN function, the distance computation was based on an extension of the Gower distance [28]. For continuous variables, we used the median to give a central measurement for the 5 nearest neighbors that were used to impute a missing value. For categorical variables, we used the mode to impute [27]. We used RStudio (version 4.0.3; R Foundation for Statistical Computing) as a programming language.

Further handling of missing data, including data imputation and the handling of outliers, as well as other used software packages, are described in Multimedia Appendix 2 [27,29-41].

##### Identification of Patient Clusters

To assign patients to clusters, we performed a K-means cluster algorithm. By using the K-means algorithm after data cleaning, we clustered individual cases into a k number of clusters using the squared Euclidean distance variable [42]. We minimized the distance between so-called centroids (1 centroid for each cluster) and the objects of each cluster. To evaluate the result of the K-means algorithm (number of clusters), we used the silhouette coefficient (SC), which measures the cohesion and segregation of each data point [43]. The closer the SC value gets to 1, the stronger the cohesion of data points within 1 cluster and the segregation between data points within 1 cluster relative to data points in another cluster. We determined the optimal number of patient clusters by the highest SC value for each diagnosis group.

##### Visualization and Description of Patient Clusters

**Figure 1:**
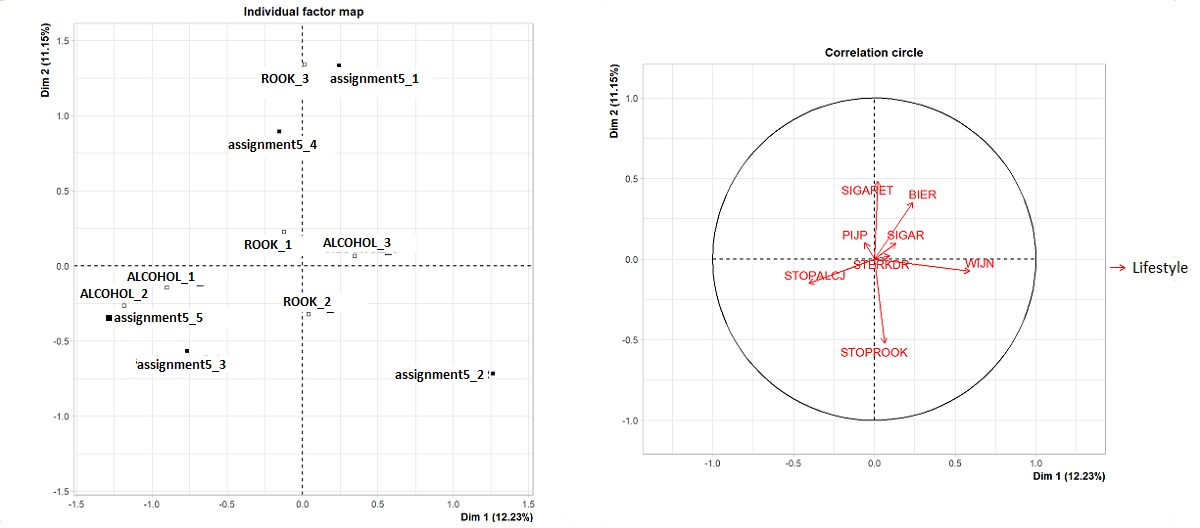
Multiple factor analysis plot. WIJN: Glasses of wine consumed per week; BIER: glasses of beer consumed per week; STERKDR: glasses of liquor consumed per week; SIGARET: number of cigarettes smoked per day; SIGAR: number of cigars smoked per day; PIJP: number of packages of pipe tobacco smoked per week; STOPALCJ: time since stopped drinking in years; STOPROOK: time since stopped smoking in years; ROOK_1: no, I do not smoke; ROOK_2: no, I do not smoke, but I used to; ROOK_3: yes, I do smoke; ALCOHOL_1: no, I do not drink alcohol; ALCOHOL_2: no, I do not drink alcohol, but I used to; ALCOHOL_3: yes, I do drink alcohol.

To enable visualization and to describe the characteristics of the identified patient clusters, we employed MFA [44]. Since the patient clusters consisted of quantitative and qualitative variables, we applied a factorial method to visualize the mutual relationships of the variables. We mapped quantitative variables by using the correlation circle based on principal component analysis. Qualitative variables, as well as cluster numbers, were visualized by using the individual factor map [45]. We grouped positively correlated variables in a correlation circle, which was visualized by arrows that lie together in the same direction in the correlation circle. Negatively correlated variables were presented opposite of each other. The further away the variables lay from the center of the correlation circle, visualized by longer arrows, the better these variables were represented within the concept. A particular topic is assessed by a few questions, which together illuminate a concept. For example, perception is a concept that is elucidated by 8 items of the Brief Illness Perception Questionnaire. For each concept, we performed this MFA analysis based on the prostate and colorectal cancer data (Figure 1, Multimedia Appendix 3).

To standardize, we used a cutoff point of 0.5 for the quality of the projection of a variable on 1 of the dimensions in the correlation circle. The same threshold was applied for the individual factor map when describing the characteristics of the clusters. We accounted for the variables drawn above these thresholds.

The variables that clustered together based on these procedures were described in different patient clusters for colorectal cancer and prostate cancer separately.

### Part 2: Usability Study

#### Design

To assess the clinical usability of the identified patient clusters, we applied a qualitative approach by conducting expert panel group interviews. Due to the COVID-19 pandemic, the group interviews were held online.

#### Study population

Both patients with cancer and professionals formed the panel of experts. Eligible health care professionals were professionals from various care disciplines with expertise in the field of oncology, including prostate or colorectal cancer. Eligible participants for the patient-expert panel were adult former and current patients with colorectal or prostate cancer who completed primary cancer treatment and may still receive adjuvant therapy. Other inclusion criteria included having basic computer skills, internet access, and a digital device with a camera and speakers.

#### Procedure and Data Collection

Through an information letter, we recruited potential participating health care professionals from 2 regional hospitals: a GP society and an oncology physiotherapy network. These professionals approached other eligible health professionals and patients (snowball sampling). The researchers assessed the eligibility criteria, and detailed information was offered by phone. All participants provided informed consent before enrollment in the study.

We interviewed the professional expert panel, the expert panel of patients with colorectal cancer, and the expert panel of patients with prostate cancer separately. We held semistructured group interviews based on a topic list (Multimedia Appendix 4) with a maximum duration of 120 minutes to gain insight into the potential clinical usability of the identified patient clusters, as assessed by the health care professionals and patients with cancer. The group interviews followed a fixed structure. After a short introduction of the project, in which the purpose of the meeting was explained again, the patient clusters were presented to the panel, and the following topics were discussed: (1) the number of the patient clusters and recognizability of the content; (2) the forms of cancer aftercare that best fit each cluster; (3) the usefulness, meaningfulness, and opportunities of patient clusters concerning tailor-made aftercare referral; and (4) the preconditions for implementing patient clusters in clinical practice. Prior to the group interviews, the participants received information about the patient clusters and regional cancer aftercare possibilities. Additionally, they received a brief online questionnaire to gather information about personal characteristics. The participating health care professionals also received some preparation questions.

#### Data Analysis Expert Panels

We analyzed personal characteristics descriptively. Video recordings and additional notes from the online group interviews were analyzed based on an abridged transcript. We employed content-directed analysis [46] to describe and understand the collected qualitative data systematically [47]. We coded and categorized the data based on the structure of the topics and questions in line with the topic list. Three researchers (Pieter Eijgenraam, Alina Kramme, and author IMK) independently performed the coding and categorizing. To increase trustworthiness, 4 researchers (Willem Emons, Roy Jorissen, Pieter Eijgenraam, and author IMK) reviewed the codes and categories and reached an agreement on the results [48]. Subsequently, the participants received a summary of the key points for verification of the content (member check).

## Results

### Part 1: Patient Clusters

In total, 3989 colorectal cancer cases (1371 participants in the 2009 colorectal wave and 2618 participants in the 2010 colorectal wave) and 696 prostate cancer cases were included in the cluster analysis (Table 1). Participants varied in age between 29 and 85 years. A description of all characteristics is provided in Multimedia Appendix 5 [19,21,22,25,49].

**Table 1:** Basic characteristics of participants with colorectal cancer (n=3989) and participants with prostate cancer (n=696).

Variable		Colorectal cancer		Prostate cancer
**Gender, n (%)**				
	Male	2220 (55.6)	696 (100)	
	Female	1769 (44.4)	0 (0)	
**Age (years), mean (SD)**				
	At the time of diagnosis	64.7 (9.8)	67.4 (7.3)	
	At the time of questionnaire	69 (9.6)	70.8 (7.2)	
**Marital status, n (%)**				
	Married	3011 (75.5)	586 (84.2)	
	Divorced	204 (5.1)	27 (3.9)	
	Widowed	640 (16)	65 (9.3)	
	Never married	134 (3.4)	18 (2.6)	
**Educational level, n (%)**				
	Lower education	777 (19.5)	117 (16.8)	
	Secondary education	1247 (31.3)	162 (23.3)	
	Secondary vocational education	1179 (29.6)	249 (35.8)	
	University	786 (9.7)	168 (24.1)	
**Employment status, n (%)**				
	Yes	604 (15.1)	89 (12.8)	
	No	3385 (84.9)	607 (87.2)	
**Socioeconomic status, n (%)**				
	Low	833 (20.9)	118 (17.0)	
	Medium	1631 (40.9)	270 (38.8)	
	High	1454 (36.4)	292 (41.9)	
	Living in a nursing home	71 (1.8)	16 (2.3)	
BMI, mean (SD)		26.7 (4.2)		26.5 (3.3)
**Assigned numbering cluster, n (%)**				
	Cluster 1	1788 (44.8)	197 (28.3)	
	Cluster 2	1144 (28.7)	85 (12.2)	
	Cluster 3	1057 (26.5)	144 (20.7)	
	Cluster 4	N/A^a^	159 (22.8)	
	Cluster 5	N/A	111 (16)	

^a^N/A: not applicable.

#### Identification of Patient Clusters

**Figure 2:**
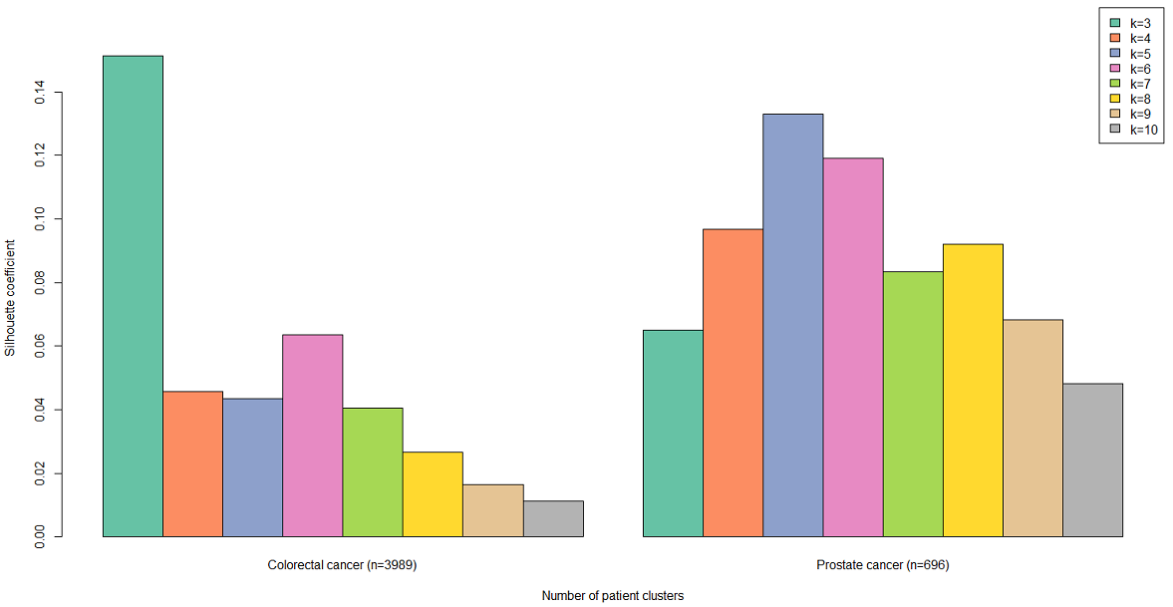
Silhouette coefficients per diagnosis group and number of clusters.

We calculated the highest SC value within the prostate cancer sample for 5 patient clusters and the highest SC value within the colorectal cancer sample for 3 patient clusters (Figure 2).

The main distinguishing characteristics of the patient clusters are described in Table 2.

**Table 2:** Main characteristics of the patient clusters for colorectal cancer (n=3989) and prostate cancer (n=696).

Patient cluster	Colorectal cancer	Prostate cancer
Patient cluster 1	Have a higher socioeconomic status Have a lower BMI More patients were diagnosed some time ago Drink alcohol more often, mainly wine More patients who exercise or do sports Lower stage of disease Do not frequently have an appointment with the specialist and have no need for one Have the fewest comorbidities Sense a small effect on their lives because of their illness More likely to think that their illness will not last long, have a sense of control, and are confident that the treatment will work Have a high understanding of their disease Recognize fewer symptoms and worry less about their illness Experience a small emotional effect Score high on the functioning scales, including the highest on emotional functioning and quality of life.	Younger Relatively higher education but not the highest education More often have a paid job More smokers Tend to drink alcohol more often Do not feel well informed, are less satisfied with the information they receive, and find that information less helpful Use the internet more often to find information about their disease.
Patient cluster 2	Lower socioeconomic status Have a higher BMI More often elderly patients who are widows or widowers More often have lower education More patients who have been diagnosed with their disease a shorter time ago More often deceased Tend to represent fewer alcohol users and smokers Least active in terms of exercise Have most often a higher stage of the disease Visit the general practitioner and cancer specialist more often Discussed coming back more often Have a higher number of comorbidities Problems with personality and fatigue on a physical and mental level and more characterized by anxiety and depression More likely to report a high degree of impact on their lives; think the illness will last longer Indicate a lower level of control Experience many symptoms Have a high degree of concern about their illness Feel an extreme effect on an emotional level Have reasonable confidence in the success of their treatment Score lower on the functioning scales Score high on fatigue, breath shortness, insomnia, pain, loss of appetite, nausea, and vomiting	Younger More often have higher education Higher socioeconomic status Lower stage of disease Tend to drink alcohol more often, even more than cluster 1 More liver problems Understand their illness better and have more confidence in their treatment Higher scores on physical, emotional, and social scales and lower scores on fatigue and pain Feel better informed and have less need for more information about their disease Use the internet more often to find information about their disease.
Patient cluster 3	Younger More often divorced Higher representation of middle socioeconomic status and people who live in an institution More often patients who have a job Drink alcohol more often More patients who exercise or do sports Have a higher stage of disease compared to cluster 1 More often have an appointment with the specialist regarding cancer and have also discussed returning to the specialist more often compared to cluster 1 Have fewer comorbidities, but depression is more common Relatively fewer problems with personality, fatigue, and depression compared to cluster 2 Relatively more fears and more negative affectation compared to cluster 1 Have a more neutral perception of their disease Not very distinctive on quality of life	Lower education Lower socioeconomic status Do household tasks more often More often stopped drinking alcohol More comorbidities Have a more negative self-image, feel a greater impact on their lives and emotions, and are more concerned Lower score on the physical, emotional, and social scales and higher score on fatigue and pain Do not feel well informed, are less satisfied with the information they receive, and find that information less helpful
Patient cluster 4	N/A^a^	Higher education but not the highest More often have an advanced stage of disease More often deceased More often disabled due to their disease More often stopped drinking alcohol More comorbidities Have a more negative self-image, illness has a greater impact on their lives and emotions, and are more concerned Lower score on the physical, emotional, and social scales and higher score on fatigue and pain.
Patient cluster 5	N/A	Lower education Lower socio-economic status More often stay in a nursing home More often without a partner More often stopped drinking alcohol Understand their illness better and have more confidence in their treatment Use the internet less often to find information about their disease.

^a^N/A: not applicable.

#### Visualization and Description of Patient Clusters

We described participant characteristics of 5 clusters of patients with prostate cancer and the 3 colorectal cancer clusters in Table 2 based on the MFA analysis. Not all the same concepts were measured in the different data sets available (ie, colorectal data and prostate data), as displayed in Table 1. As a result, certain concepts could not be reflected in the clusters.

### Part 2: Usability Study

#### Expert Panel Participants

A total of 23 people participated in this part of the study (Table 3). Of the 8 patient experts approached, 6 (75%) filled in the brief online questionnaire, with 3 (50%) for prostate cancer and 3 (50%) for colorectal cancer. Moreover, 5 (83.3%) took part in the group interviews. Reasons for not participating included not wanting to participate digitally (1/6, 16.7%) and an emergency medical appointment (1/6, 16.7%). One (16.7%) person did not state a reason. Of the 20 professional experts approached, 17 (85%) participated. Reasons for nonparticipation were maternity leave (1/20, 5%), no time (1/20, 5%), and unknown (no response, 1/20, 5%).

**Table 3:** Characteristics of expert panel participants (N=23).

Characteristic	Patient experts (n=6)	Professional experts (n=17)
Female gender, n (%)	1 (16.7)	13 (76.5)
Age, median (min-max)	60 (48-79)	48 (33-64)
Prostate cancer diagnosis, n (%)	3 (50)	N/A^a^
Colorectal cancer diagnosis, n (%)	3 (50)	N/A
Time since diagnosis, median (min-max)	2.8 (1-8)	N/A
Cancer detected during control visit, n (%)	2 (33.3)	N/A
Nurse specialist hospital, n (%)	N/A	2 (11.8)
Nurse specialist general practice, n (%)	N/A	2 (11.8)
General practitioner, n (%)	N/A	2 (11.8)
Internist oncologist, n (%)	N/A	2 (11.8)
Psychologist, n (%)	N/A	2 (11.8)
Oncology physiotherapist, n (%)	N/A	2 (11.8)
Oncology surgeon, n (%)	N/A	1 (5.9)
Rehabilitation physician, n (%)	N/A	1 (5.9)
Complementary health therapist/lifestyle coach, n (%)	N/A	1 (5.9)
Acupuncturist, herbalist, n (%)	N/A	1 (5.9)
Staff advisor oncology, n (%)	N/A	1 (5.9)
Years of work experience (oncology), median (min-max)	N/A	15 (0.5-40)
Cancer aftercare provider, n (%)	N/A	14 (82.4)

^a^N/A: not applicable.

#### Expert Panel Interviews

In total, 7 group interviews took place. We conducted 1 group interview with patients with prostate cancer (3/5, 60%) and 1 with patients with colorectal cancer (2/5, 40%). Five professional expert panel group interviews took place in varying compositions regarding the profession and with a group size of 3 to 5 participants. One individual interview was conducted.

#### Clinical Usability of the Patient Clusters

Most of the participants recognized the clustering as distinctive “profiles,” and all variables described were assessed as important factors regarding tailored referral to aftercare. They indicated that the variables follow a certain hierarchy that should be accounted for when considering referral to appropriate aftercare. The expert panel stated that describing the clusters in tabular form with many variables outlined in the text was too difficult to oversee. Moreover, participants were concerned that patients would be placed into fixed categories by using this tabular format. Furthermore, a conversation with patients would be necessary to clarify their support needs. The clusters could also serve as a valuable starting point and guidance for this conversation because they provide meaningful content and structure.

Care providers often don't look beyond their specialism. A broad view is missing. Other fields should also be considered in the conversation about aftercare.

Patient with prostate cancer

Therefore, participants suggested the development of a conversation tool that could provide insight into the content and structure of these clusters. To guide professionals through the hierarchy of variables, a decision tree could be integrated into this tool. In addition, participants suggested that access to information about available aftercare initiatives should be made available. This would also ensure a good overview and seemed to be a precondition for finding suitable aftercare.

As a patient, you don't know what the disease entails and what you can expect, so you don't know what aftercare you need. You need to be well informed; only then do you know what you need.

Patient with colorectal cancer

You are very much searching and constantly retelling your whole story. It would be nice to have a choice of presorted relevant options of aftercare. The disease already costs you a lot of energy. Searching also takes a lot of energy!

Patient with colorectal cancer

The tool content should be comprehensive, clearly structured, and easy to use. The patient, not the professional or the application, should always make the final decision on aftercare. The professional experts also wished to link existing data from the electronic patient files to the decision tool.

Using a decision aid based on the patient clusters would be a good tool for care providers to gain a better understanding and to get an overview when it comes to referral to the right aftercare.

Patient with prostate cancer

This kind of tool could take the administrative burden off the nurses’ shoulders.

Oncology specialist

## Discussion

### Principal Findings

This study aimed to (1) identify, visualize, and describe patient clusters within colorectal and prostate cancer populations and (2) explore the potential usability of the patient clusters in clinical practice to improve referral to cancer aftercare.

We identified, described, and presented 5 patient clusters among a prostate cancer population and 3 patient clusters among a colorectal cancer population to an expert panel for evaluation.

Most notably, by performing the cross-sectional data analysis, we included all available variables in the data sets without any human preselection, and the number of patient clusters was solely determined by the SC. Our approach to cluster the data of individuals based on their characteristics is consistent with clinical practice, wherein an oncology professional encounters a patient with individual characteristics. In our results, easily detectable characteristics such as age, employment status, and socioeconomic status clustered with less easily recognizable characteristics, such as illness perception. This interrelationship between different characteristics can support health care providers in the conversation with patients for referral to appropriate follow-up care.

Contrary to our method, de Rooij et al [50] explored the relation of symptoms among a selection of PROFILES registry variables in their network analysis, such as the European Organization for Research Treatment of Cancer Quality of Life Questionnaire (EORTC QLQ-C30) symptom scales and emotional and cognitive functioning scales). Noticeably, however, our results for colorectal cancer data are in line with the findings of de Rooij et al [50] regarding the corresponding variables (eg, fatigue, pain, dyspnea, sleeping problems, appetite loss, and nausea and vomiting), which might strengthen our findings.

Professional and patient experts considered the insight that different subgroups can be distinguished within 1 diagnosis group and can be valuable for referring patients to the appropriate aftercare. Participants largely recognized the classification into the clusters. However, the expert panel deemed the way of presenting the clusters in textual tabular form to be unpractical for routine care. To have a meaningful conversation about referral to appropriate aftercare, professionals and patients would like to have guidance to help them discuss relevant topics, which then can lead to the most suitable choices for cancer aftercare. Therefore, a complete overview of current aftercare initiatives is also needed. The experts suggested developing a digital decision and referral aid based on the patient clusters to detect a patient's support needs and risks and link them to the available aftercare options.

Overall, this study succeeded in identifying patient clusters that are also seen in routine care and recognized by health care professionals. Our results show that this holistic, explorative machine-learning approach can provide a foundation to identify clinically meaningful patient clusters. Consequently, our results can serve as a first step to improve referrals to cancer aftercare in daily practice, which is in line with the goals of the Taskforce Cancer Survivorship [8,10].

### Limitations

Like all research, this study has its limitations. Participant data were not highly distinguishable for all variables because not all answer options were distinguishable (ie, the distinguishing variables had a lot of overlap and were therefore not good indicators for distinguishing between clusters). This problem could technically be solved by using a larger number of patient clusters. However, this would be less appropriate for clinical use because a larger number of clusters makes it difficult for professionals to get an overview of the clusters.

The data from the PROFILES registry were generated about 10 years ago, while we retrieved the data from the qualitative study in 2020. However, we do not expect a negative impact from this time difference, as we assume that patients with cancer are not significantly different now than they were 10 years ago.

Finally, we interviewed mainly professional and patient experts, but patient experts’ opinions were relatively underrepresented. Consequently, we may not have achieved data saturation.

### Future Directions

Since the identification and use of patient clusters among colorectal and prostate cancer populations are still in their infancy, future research should further focus on identifying distinguishing key variables to optimize the number and content of patient clusters. Building upon a data-driven approach, an additional expert-driven approach could provide a qualitative improvement in the selection of variables. Both patient and professional experts should be equally involved in this process. Researchers should explore in what form a digital referral aid could be of added value in clinical practice. Our results might provide valuable insights as a basis for the development of smart referral technology.

Furthermore, identifying longitudinal patient patterns, based on data gathered over time, might be the next step to generate insights into the course of a patient’s situation and deviations from “expected recovery.” The process of identifying patient patterns could be automated by creating a data tunnel linked to electronic patient records and by automatically generating trend analyses that could provide insights into the development of an individual’s disease and recovery over time.

### Conclusions

This study demonstrates that a fully data-driven approach can be used to identify distinguishable and recognizable patient clusters in large data sets within colorectal and prostate cancer populations. Using patient clusters based on their characteristics can be supportive for health professionals in the aftercare conversation. Patient clusters integrated into a smart digital conversation and referral tool might be an opportunity to improve the referral to cancer aftercare.

## Appendix

Multimedia Appendix 1 Variables included in the cluster analysis available from the PROFILES (Patient-Reported Outcomes Following Initial Treatment and Long-term Evaluation of Survivorship) registry.

Multimedia Appendix 2 Handling missing data including imputation and handling outliers and used software packages.

Multimedia Appendix 3 The interpretation of the multiple factor analysis.

Multimedia Appendix 4 Topic list for the expert panel interview.

Multimedia Appendix 5 Remaining characteristics of participants with colorectal cancer (n=3989) and participants with prostate cancer (n=696).

## Abbreviations

EORTC QLQ-C30: European Organization for Research Treatment of Cancer Quality of Life Questionnaire
GP: general practitioner
KNN: K-nearest neighbor
MFA: multiple factor analysis
PROFILES: Patient-Reported Outcomes Following Initial Treatment and Long-term Evaluation of Survivorship
SC: silhouette coefficient

## Data Availability

Data from the PROFILES (Patient-Reported Outcomes Following Initial Treatment and Long-term Evaluation of Survivorship) registry are freely available for noncommercial scientific research, subject to the study question, privacy and confidentiality restrictions, and registration [15].
