# Supplementary material for: Identification and Potential Use of Clusters of Patients With Colorectal Cancer and Patients With Prostate Cancer in Clinical Practice: Explorative Mixed Methods Study
Source: JMIR Cancer. 2022 Dec 27;8(4):e42908. doi: 10.2196/42908 (PMC9832354; doi:10.2196/42908)
Supplement: Multimedia Appendix 1 [file cancer_v8i4e42908_app1.docx]

**Appendix 1.** Variables included in the cluster analysis available from PROFILES.

Lifestyle was assessed by questioning, tobacco and alcohol use, and comorbidity were assessed by using the Comorbidity questionnaire [18]. To assess the cognitive and emotional representations of illness, eight items of the Brief Illness Perception Questionnaire (BIPQ) were included [19]. Clinical and cancer-related data were used which originated from the Netherlands Cancer Registry [16], comprising the time since diagnosis, age at the time of diagnosis, age at the time of filling out the questionnaire, body mass index (BMI), TNM tumor classification, vital status, cancer treatment (eg, surgery, systemic treatment, radiotherapy, hormonal therapy, no treatment, treatment unknown). Various items were used to assess the utilization of cancer care [20]. Data on health-related quality of life were used by including all subscales from the EORTC-QLQ-C30 [21]. This data was not available in the 2009 dataset of colorectal cancer patients. The following data was only present in both colorectal cancer samples: data on physical activity, type D personality (DS-14 subscales negative affectivity and social inhibition) [22,23], fatigue including the subscales physical fatigue and mental fatigue from the Fatigue Assessment Scale (FAS) [24], anxiety and depression including the two subscales for anxiety and depression of the Hospital Anxiety and Depression Scale (HADS) [25]. From only the prostate cancer dataset, data from the EORTC QLQ-INFO26 concerning the perception of received information could be used [26].
