# Supplementary material for: Identification and Potential Use of Clusters of Patients With Colorectal Cancer and Patients With Prostate Cancer in Clinical Practice: Explorative Mixed Methods Study
Source: JMIR Cancer. 2022 Dec 27;8(4):e42908. doi: 10.2196/42908 (PMC9832354; doi:10.2196/42908)
Supplement: Multimedia Appendix 2 [file cancer_v8i4e42908_app2.docx]

**Appendix 2.** Handling missing data including imputation and handling outliers and used software packages.

#### Handling missing data

The data analyses on colorectal and prostate cancer samples were conducted separately. The two colorectal cancer samples were merged and all data were assessed for outliers and aberrant measurement data. Complete data sets are an important precondition for performing the cluster analysis and therefore non-responders and variables with more than 50% missing data (non-available’s; NA’s) were removed.

NAs were imputed conform recommendations of Kalton & Kasprzyk [29] and Rubin [30] by using the nearest neighbor imputation (NNI) technique which is appropriate to apply for survey data with a high number of respondents [31,32]. In this study, the fife nearest neighbors-imputation technique was applied which is derived from the NNI by using donor observations of the actual data [27,33], with nearest defined by a distance function of the auxiliary variables [34]. This imputation method is applicable for samples with multiple missing values and suitable for both discrete and continuous variables [35]. This method leads to a consistent imputation that is based on all included variables.

#### Handling outliers

To downsize the effects of large size variables (or having a great variability) on cluster analysis, several standardization methods were conducted [36]. To accommodate extreme outliers in continuous variables (except for BMI) Winsorized Trimming was conducted which replaces outliers on the high side (and low side) by the next value to the highest (respectively lowest) value within the boundary of the outer fence [37]. To highlight values that are considered to be extreme outliers the outer fences are set to three times the interquartile range [37]. Continuous variables were standardized with a z-score-standardization method for normally distributed variables, whereas skewed data is standardized with a Min-Max-standardization method. For nominal or ordinal variables with categories that had a lower number of objects, lower than the square root of the total number of objects, the categories were aggregated with the nearest category to the category with low numbers. Subsequently, these variables were standardized using dummy variables.

#### Removing near-zero variance variables

Variables with a near-zero variance were removed because they do not contribute information and therefore the minority of the values that are represented in a near-zero variable could have an undue influence on the model [38].

##### ***Used Software packages***

- FactoMineR, used to exploratory analyze the data with respect to identifying hidden patterns in the dataset. In particular to use the MFA for variables structured in groups [39].
- Factoextra, used to create and visualize the output of multivariate data analyses with Multiple Factor Analysis (MFA) [40].
- Provides ggplot2 Cluster, used to cluster the data with the K-means algorithm.
- MASS, used to support Venables and Ripley [41].
- VIM package, used to impute missing data with the use of the KNN method [27].
