# Supplementary material for: Identification and Potential Use of Clusters of Patients With Colorectal Cancer and Patients With Prostate Cancer in Clinical Practice: Explorative Mixed Methods Study
Source: JMIR Cancer. 2022 Dec 27;8(4):e42908. doi: 10.2196/42908 (PMC9832354; doi:10.2196/42908)
Supplement: Multimedia Appendix 3 [file cancer_v8i4e42908_app3.docx]

**Appendix 3.** The interpretation of the MFA.

In the correlation circle on the right-hand side in figure 1, derived from the prostate cancer data, the relationship between variables considering the concept: lifestyle, in terms of how many glasses of wine do you drink a day and how many cigarettes do you smoke a day (amongst other questions, see table 1), the quality of the representation and the correlation between these variables and the dimensions are shown. The first dimension mostly correlates positively with wine consumption as does the second dimension with cigarette consumption, positioned opposed to this the time since a participant quit smoking is shown and is the variable that correlates negatively with the second dimension.

In the plot on the left-hand side (figure 1) the qualitative variables considering the concept: of lifestyle are shown. Participants who smoke (ROOK_3) have positive coordinates on the second axis along with participants that are clustered in clusters 1 and 4 (assignment5_1 and assignment5_4), thus cluster 1 can be looked at as the group where the number of participants who smoke is more represented. In cluster 2 (assignment5_2), people who drink more wine are mainly represented, at the same time, these are the people who have more often stopped smoking for a longer time ago, both variables score low on the second dimension. Cluster 5 (assignment5_5) often includes participants who have stopped drinking alcohol (ALCOHOL_2) or who indicate that they do not drink alcohol at all (ALCOHOL_1). For clusters 3 and 4 this picture is not so clear.

Most of the other qualitative or quantitative variable categories are close to the origin. This indicates that these categories are not related to the first or second dimension.
