# Supplementary material for: Identification and Potential Use of Clusters of Patients With Colorectal Cancer and Patients With Prostate Cancer in Clinical Practice: Explorative Mixed Methods Study
Source: JMIR Cancer. 2022 Dec 27;8(4):e42908. doi: 10.2196/42908 (PMC9832354; doi:10.2196/42908)
Supplement: Multimedia Appendix 4 [file cancer_v8i4e42908_app4.docx]

**Appendix 4.** Topic list expert-panel interview.

1. The recognizability of content and number of the patient clusters
   1. In your opinion, to what extent are the characteristics described recognizable as important characteristics for referral to aftercare?
      1. Would you consider adding or removing certain characteristics?
   2. To what extent do you recognize the composition of characteristics of the clusters from your own experience?
      1. Would you consider merging certain profiles? If so, which ones?
      2. Would you consider adding a completely different profile? If so, what are the characteristics that this new profile should comprise?
   3. In your opinion, which characteristics within a profile are crucial in the choice for suitable aftercare?

1. Linking cancer aftercare possibilities to patient clusters (provided with an overview of all available options in the region)
   1. Looking at cluster x and imagining a patient who fits this cluster, what form(s) of cancer aftercare could be appropriate for this patient, in your opinion?

1. Usability and opportunities of patient clusters in health care practice
   1. To what extent are patient clusters useful when it comes to improving referral to cancer aftercare?
   2. In what way could the patient clusters actually be used in clinical practice?

1. Preconditions for implementing patient clusters in clinical practice
   1. What are the essential preconditions for implementing patient clusters in healthcare practice?
   2. What kind of barriers do you expect?
   3. In which particular ways do you think patient clusters can be successfully brought into clinical practice?
