# Supplementary material for: Identification and Potential Use of Clusters of Patients With Colorectal Cancer and Patients With Prostate Cancer in Clinical Practice: Explorative Mixed Methods Study
Source: JMIR Cancer. 2022 Dec 27;8(4):e42908. doi: 10.2196/42908 (PMC9832354; doi:10.2196/42908)
Supplement: Multimedia Appendix 5 [file cancer_v8i4e42908_app5.docx]

**Appendix 5.** Remaining characteristics of colorectal cancer (N = 3989) and prostate cancer (N = 696) participants.

| **Variable** | **Category** | Colorectal cancer | Prostate cancer |
| --- | --- | --- | --- |
|  |  |  |  |
| **Stage disease, N (%)** |  |  |  |
|  | 1  2  2A  2B  3  3A  3B  3C  4  X  [ 2 others ]  1  2  3  4 | 1060 (26.6)  295 (7.4)  1097 (27.5)  123 (3.1)  215 (5.4)  136 (3.4)  585 (14.7)  240 (6.0)  187 (4.7)  39 (1.0)  12 (0.3)    N/A  N/A  N/A  N/A | N/A^a^  N/A  N/A  N/A  N/A  N/A  N/A  N/A  N/A  N/A  N/A  2 (0.3)  497 (71.4)  132 (19.0)  65 (9.3) |
| **Vital status, N (%)** |  |  |  |
|  | Alive  Deceased | 2937 (73.6)  1052 (26.4) | 560 (80.5)  136 (19.5) |
| **Time since diagnosis in years, M^b^ (SD^c^)** |  |  |  |
|  |  | 4.8 (2.7) | 4 (1.2) |
| **Treatment (KR data), N (%)** |  |  |  |
|  | Surgery | 3946 (98.9) | 197 (28.3) |
|  | Radiotherapy | 1094 (27.4) | 273 (39.2) |
|  | Systemic therapy | 1193 (29.9) | 0 (0.0) |
|  | Hormonal therapy | 3 (0.1) | 209 (30.0) |
|  | No therapy or active surveillance | 4 (0.1) | 130 (18.7) |
| **Number of consults in the past 12 months, M (SD)** |  |  |  |
|  | General practitioner  General practitioner, due to cancer  Specialist  Specialist due to cancer | 4 (6.1)  1.2 (3.5)  1.1 (4.8)  3 (4.3) | 3.4 (2.9)  N/A  1.2 (2.1)  2.2 (1.6) |
| **Still follow up**  **Appointments, N (%)** |  |  |  |
|  | Yes  No | 3149 (78.9)  840 (21.1) | 655 (94.1)  41 (5.9) |
| **Discussed with specialist**  **how often to come back from this moment on, N (%)** |  |  |  |
|  | Yes, every 3 months  Yes, every 4 months  Yes, every 6 months  Yes, once a year  Yes, every 2 years  No | 473 (11.9)  142 (3.6)  1574 (39.5)  950 (23.8)  327 (8.2)  523 (13.1) | 74 (10.6)  26 (3.7)  360 (51.7)  200 (28.7)  4 (0.6)  32 (4.6) |
| **Comfortable with**  **follow up scheme, N (%)** |  |  |  |
|  | Yes  No, want more follow up  No, want less follow up  No, want no follow up | 3567 (89.4)  218 (5.5)  73 (1.8)  131 (3.3) | 637 (91.5)  29 (4.2)  14 (2.0)  16 (2.3) |
| **Received cancer aftercare, N (%)** |  |  |  |
|  | Received aftercare overall | N/A | 349 (50.1) |
|  | Psychologist | N/A | 20 (2.9) |
|  | Sexologist | N/A | 6 (0.9) |
|  | Social worker | N/A | 3 (0.4) |
|  | Pastoral worker | N/A | 1 (0.1) |
|  | General practitioner | N/A | 42 (6.0) |
|  | Dietitian | N/A | 12 (1.7) |
|  | Physiotherapist | N/A | 108 (15.5) |
|  | Recovery group program | N/A | 10 (1.4) |
|  | Creative therapy | N/A | 2 (0.3) |
|  | Oncological nurse | N/A | 16 (2.3) |
|  | Contact with fellow patients/survivors | N/A | 7 (1.0) |
|  | Others | N/A | 43 (6.2) |
| **Comorbidities, N (%)** |  |  |  |
|  | Heart condition | 727 (18.2) | 141 (20.3) |
|  | Stroke | 96 (2.4) | 18 (2.6) |
|  | High Blood pressure | 1288 (32.3) | 223 (32.0) |
|  | Long disease | 399 (10.0) | 80 (11.5) |
|  | Diabetes | 536 (13.4) | 94 (13.5) |
|  | Ulcer | 52 (1.3) | 10 (1.4) |
|  | Kidney disease | 134 (3.4) | 19 (2.7) |
|  | Liver disease | 124 (3.1) | 2 (0.3) |
|  | Anemia | 127 (3.2) | 29 (4.2) |
|  | Thyroid disease | 189 (4.7) | 16 (2.3) |
|  | Depression | 250 (6.3) | 43 (6.2) |
|  | Arthritis | 988 (24.8) | 156 (22.4) |
|  | Backache | 994 (24.9) | 171 (24.6) |
|  | Rheumatism | 238 (6.0) | 45 (6.5) |
| **Number of hours paid job, M (SD)** |  |  |  |
|  |  | 4.5 (11.7) | 4.1 (11.9) |
| **Unable to work, due to cancer, N (%)** |  |  |  |
|  | Not applicable  I was always able to work  I wasn’t able to work | N/A  N/A  N/A | 624 (89.7)  18 (2.6)  54 (7.8) |
| **Number of hours unable to work per week, M (SD)** |  |  |  |
|  |  | N/A | 1 (4.1) |
| **Employment status, N (%)** |  |  |  |
|  | Having a job  Pension/early retirement  Scholar/student  Unemployed  Disabled  Managing the household  Other | 604 (15.1)  2819 (70.7)  1 (0.0)  40 (1.0)  226 (5.7)  220 (5.5)  79 (2.0) | 89 (12.8)  558 (80.2)  0 (0.0)  6 (0.9)  29 (4.2)  3 (0.4)  11 (1.6) |
| **Disability percentage, M (SD)** |  |  |  |
|  |  | 3.5 (18) | 2.9 (16.3) |
| **Disability due to the disease, N (%)** |  |  |  |
|  | NA  Yes  No | 3789 (95.0)  130 (3.3)  70 (1.8) | 674 (96.8)  5 (0.7)  17 (2.4) |
| **Smoking, N(%)** |  |  |  |
|  | No  No, but I used to  Yes | 1284 (32.2)  2267 (56.8)  438 (11.0) | 154 (22.1)  459 (66.0)  83 (11.9) |
| **Time since stopped smoking in years, M (SD)** |  |  |  |
|  |  | N/A | 16.1 (16.4) |
| **Number of cigarettes per day, M (SD)** |  |  |  |
|  |  | 1.3 (4.8) | 1.1 (4.3) |
| **Number of cigars per week, M (SD)** |  |  |  |
|  |  | 0.5 (4.7) | 0.5 (4.1) |
| **Number of packages of pipe tobacco per week, M (SD)** |  |  |  |
|  |  | 0 (0.1) | 0 (0.3) |
| **Alcohol consumption, N (%)** |  |  |  |
|  | No  No, but I used to  Yes | 1055 (26.5)  361 (9.0)  2573 (64.5) | 90 (12.9)  84 (12.1)  522 (75.0) |
| **Time since stopped drinking in years, M (SD)** |  |  |  |
|  |  | N/A | 1.3 (5.4) |
| **Glasses/consumption per week, M (SD)** |  |  |  |
|  | Beer  Wine  Liquor | 1.7 (4.7)  2.7 (5.1)  0.9 (3.1) | 2.8 (5.2)  3.2 (5.4)  1.4 (3.5) |
| **Physical Activity, hours per week, M (SD)** |  |  |  |
|  | Walking summer  Walking winter  Biking summer  Biking winter  Gardening summer  Gardening winter  Household summer  Household winter | 5.2 (5.4)  3.9 (4.6)  4.9 (7)  2 (3.5)  3 (4.7)  0.7 (1.6)  7.9 (10.1)  7.7 (10.1) | N/A |
| **Weekly sporting**  **activities in the past year, N (%)** |  |  |  |
|  | No  Yes | 2674 (67.0)  1315 (33.0) | N/A |
| **Type –D personality (DS-14)^d^, M (SD)** |  |  |  |
|  | Negative affectivity | 7.3 (6.2) | N/A |
|  | Social Inhibition | 7.9 (6.2) | N/A |
| **Illness Perception (BIPQ)^e^, M (SD)** |  |  |  |
|  | Affect on life | 3.9 (2.6) | 3.7 (2.5) |
|  | Time illness continues | 4.4 (3.4) | 5.7 (3.6) |
|  | Control over illness | 5.1 (3.1) | 5.3 (3.3) |
|  | Treatment helps | 7.3 (2.7) | 7.5 (2.7) |
|  | Experience symptoms | 3.4 (2.6) | 3.5 (2.6) |
|  | Concerned about illness | 4 (2.7) | 3.7 (2.7) |
|  | Understanding illness | 6.9 (2.9) | 7.4 (2.6) |
|  | Illness affects emotionally | 3.4 (2.5) | 3.3 (2.6) |
| **Fatigue (FAS)^f^, M (SD)** |  |  |  |
|  | Physical subscale | 11.6 (4.1) | N/A |
|  | Mental subscale | 9.3 (3.6) | N/A |
| **Anxiety and Depression (HADS)^g^, M (SD)** |  |  |  |
|  | Anxiety subscale | 4.7 (3.8) | N/A |
|  | Depression subscale | 4.7 (3.6) | N/A |
| **Health-Related Quality of life (EORTC QLQ-C30)^h^ , M (SD)** |  |  |  |
|  | Physical Functioning | 71.1 (21.7) | 83.1 (19.1) |
|  | Role Functioning | 74 (24.1) | 81.2 (26.7) |
|  | Emotional Functioning | 79.8 (19.8) | 87.4 (18.7) |
|  | Cognitive Functioning | 78.9 (18.7) | 84.5 (20.1) |
|  | Social Functioning | 75.4 (26.6) | 89.5 (19.4) |
|  | Global health status | 78.7 (16.3) | 77.7 (18.1) |
|  | Fatigue | 28 (21.7) | 19.9 (22.3) |
|  | Nausea / Vomiting | 10.5 (16.8) | 2.2 (9) |
|  | Pain | 21.3 (26.1) | 15.7 (24.3) |
|  | Dyspnea | 18.5 (24.9) | 15.4 (25.5) |
|  | Insomnia | 29.3 (29) | 18.4 (27.6) |
|  | Appetite loss | 8 (16.8) | 3.3 (12.5) |
|  | Constipation | 7.6 (17.6) | 6.7 (17.9) |
|  | Diarrhea | 9.3 (19.9) | 5.3 (15.8) |
|  | Financial Problems | 5.3 (16.4) | 4.5 (13.8) |
| **Information (EORTC QLQ-INFO25), M (SD)** |  |  |  |
|  | Treatment | N/A | 2.9 (1) |
|  | Disease | N/A | 53.8 (21.5) |
|  | Medical tests | N/A | 62 (27.7) |
|  | Other services | N/A | 18.8 (23.5) |
|  | Different places of care | N/A | 17.5 (29) |
|  | Things you can do to help yourself | N/A | 22.4 (29.6) |
|  | Written information | N/A | 74.7 (43.5) |
|  | On CD tape/video | N/A | 5.3 (22,4) |
|  | Satisfaction | N/A | 60.1 (27.7) |
|  | Wish for more | N/A | 25.6 (43.6) |
|  | Wish for less | N/A | 3.6 (18.6) |
|  | Helpful | N/A | 64.7 (26.2) |
| **Use of internet, N (%)** |  |  |  |
|  | Daily  Weekly  Monthly  No | N/A | 336 (48.3)  103 (14.8)  31 (4.4)  226 (32.5) |
| **Search information via the internet, N (%)** |  |  |  |
|  | Yes  No | N/A | 352 (50.6)  344 (49.4) |

Note:

^a^N/A = Not Applicable

^b^M = Mean

^c^SD = Standard Deviation

^d^Subscale used for Type-D personality; Negative affection (range: 0-28); Social inhibition (range: 0-28); type D if both N/A and SI score $\geq10$ [22]

^e^Brief Illness Perception Questionnaire (BIPQ); item score range: 0-10 [19].

^f^FAS; Subscale score range: 5-25 [49].

^g^HADS: subscale score range: 0-21 [25].

^h^EORTC QLQ-C30: item score range 0-100; Higher scores on functional scales represent higher levels of functioning and higher score for global health status represents a higher level of quality of life; high scores for the symptoms scales represent a higher level of problems [21].
